# Expert Views on COVAX and Equitable Global Access to COVID-19 Vaccines

**DOI:** 10.3389/ijph.2021.1604236

**Published:** 2021-12-31

**Authors:** Tania Manriquez Roa, Felicitas Holzer, Florencia Luna, Nikola Biller-Andorno

**Affiliations:** ^1^ Institute of Biomedical Ethics and History of Medicine, Faculty of Medicine, University of Zurich, Zurich, Switzerland; ^2^ Digital Society Initiative, University of Zurich, Zurich, Switzerland; ^3^ Bioethics Program, FLACSO, Buenos Aires, Argentina; ^4^ CONICET, Buenos Aires, Argentina

**Keywords:** ethics, policies, equitable global access, COVAX, COVID-19 vaccines, expert views

## Abstract

**Objectives:** We face the impossibility of having enough COVID-19 vaccines for everyone in the near future. This study aims to contribute to the debate on equitable global access to COVID-19 vaccines, tackling key ethical discussions and policy challenges regarding early phases of COVAX, the global cooperation mechanism for supporting fair vaccine allocation.

**Methods:** We conducted in-depth interviews with twelve experts and a literature research on academic articles, media sources and public statements. We built a data analysis matrix and conducted a thematic analysis.

**Results:** Our findings show, first, that interviewed experts who hold different views on vaccine allocation, including moderate nationalist perspectives, agree on joining a global cooperation mechanism. Second, incentives to join COVAX vary greatly among countries. Third, specific barriers to COVAX emerged in the early implementation phase. And fourth, countries might be trapped in a zero-sum game regarding the global vaccine supply.

**Conclusion:** We present findings that enrich analyses of early phases of COVAX (April 2020–21), we introduce three ethical discussions that provide a common ground for equitable access to COVID-19 vaccines, and we highlight policy challenges.

## Introduction

Safe and effective vaccines are being developed to control the coronavirus pandemic. However, vaccine production is slow and the current impossibility of having enough vaccine doses for everyone has opened the floor for a discussion on equitable global access to COVID-19 vaccines. This discussion raises ethical and policy issues. On the one hand, we need to discuss the allocation of limited vaccines in a pandemic, knowing that vaccines are an available lifesaving preventive intervention. In this regard, a pressing concern is the availability of vaccines in low and middle-income countries that often lack the means to buy vaccines through bilateral deals with manufacturers. On the other hand, to effectively control the pandemic and ultimately decrease suffering and death, we need to reduce virus circulation, prevent the spread of new virus mutations, and protect the vulnerable globally. We argue that all countries are vulnerable to COVID-19 and share the responsibility of mitigating the pandemic through collaborative efforts [1]. Moreover, ensuring equity in global vaccine allocation is a matter of self-interest for high-income countries [2, 3].

In this study we aim to contribute to the debate on equitable global access to COVID-19 vaccines by revealing ethical discussions and policy challenges regarding COVAX (COVID-19 Vaccines Global Access). COVAX is the most prominent global initiative for supporting fair vaccine allocation under a distributive scheme [4, 5]. The scheme is co-led by the Vaccine Alliance (Gavi), the Coalition for Epidemic Preparedness Innovations (CEPI), and the World Health Organization (WHO). Countries participating in COVAX are guaranteed access to a portfolio of vaccine candidates against the SARS-COV-2 virus, although countries can still buy vaccines outside the scheme.

We observe that many high-income countries have secured priority access to vaccines for their own populations through advance purchase agreements (APAs). These countries have initiated massive COVID-19 vaccine campaigns, while vaccine supply is still limited in most low- and middle-income countries. Middle-income countries are a diverse group by size, population, and income level. They make up to 75% of the global population and represent about one third of the global GDP. Despite being considered major engines of global growth, they also represent 62% of the world’s poor [6]. Low-income countries are proportionally the worst-off in terms of vaccine access, even though they may access vaccines through Gavi. Global access to vaccines is especially restricted in many middle-income countries, that are at times neither poor enough to access vaccines through funding agencies nor rich enough to negotiate APAs [7].

**FIGURE 1:**
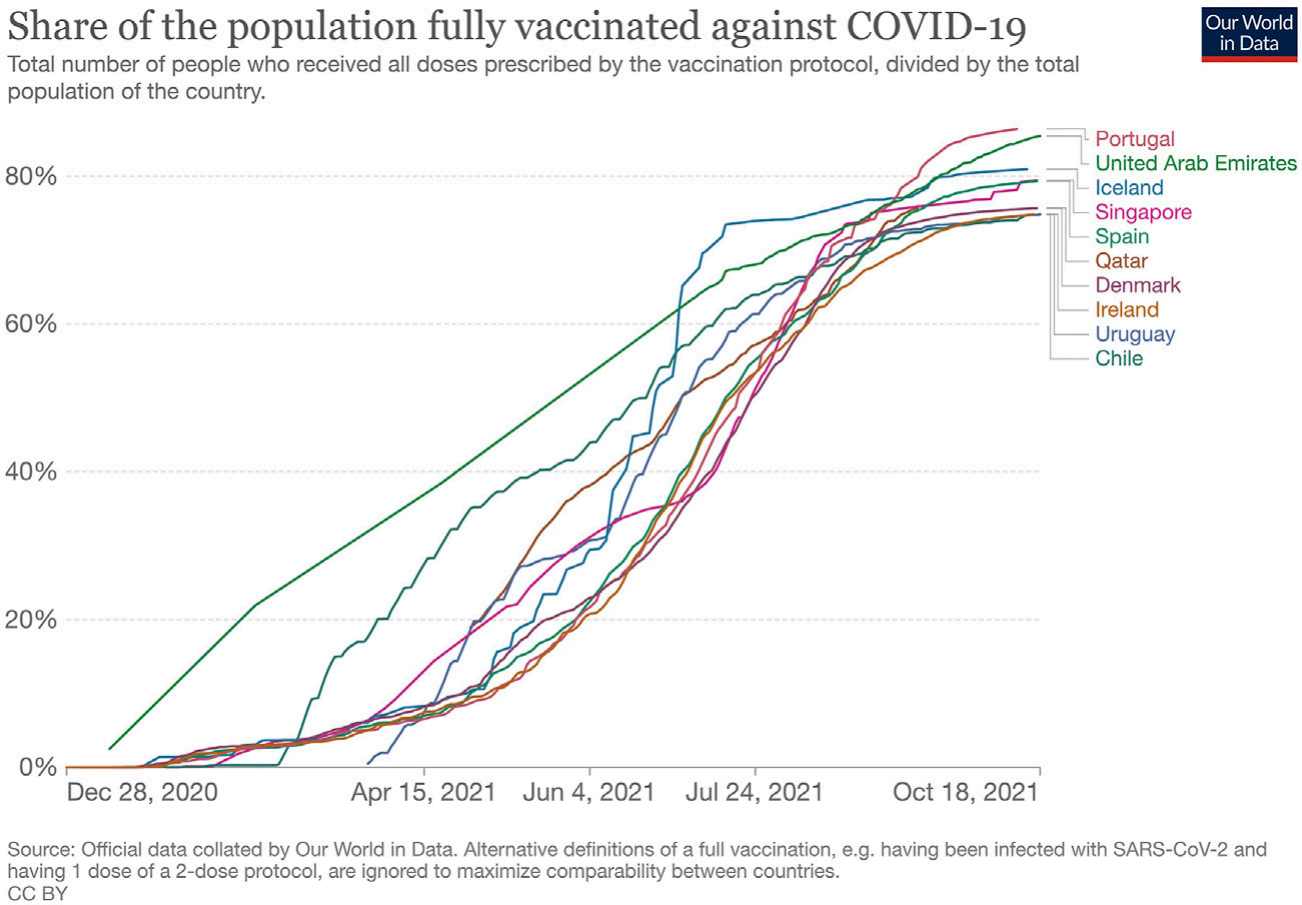
Share of the population fully vaccinated against COVID-19, Our World in Data, England and Wales, October 18, 2021.

As of October 18, 2021, the countries with higher vaccination rates are mainly those with high-income economies, according to the World Bank Classification [8]. For instance, the top 10 countries with high vaccination rates (and a population over one million), are all high-income countries [9]. Figure 1 shows the proportion of the total population that has been fully vaccinated in those countries [9].

This study is structured as follows. First, we introduce a methodological section. Second, we present the findings from expert views on key ethical and policy challenges regarding equitable access to COVID-19 vaccines, focusing on the early implementation phase of the COVAX scheme. Third, we present a critical discussion of our findings and complement them with secondary data. We conclude presenting three ethical discussions that provide a common ground for equitable access to COVID-19 vaccines, and key policy challenges that have emerged so far.

## Methods

We designed a study using mixed methodology to identify policy challenges and ethical issues regarding the COVAX scheme in the context of equitable global access to COVID-19 vaccines. Our methods include the conduct of exploratory interviews with experts and a literature research of secondary data. The purpose of using an approach that complements the findings of expert interviews and data from literature research, is to gain access to the different perspectives and aspects of global access to vaccines and COVAX. This allows us to broaden the range of issues concerning the topic rather than strengthening particular conclusions about it [10].

We carried out a literature research comprising of academic articles in journals that focus on international health, bioethics, and medicine; media reports in newspapers and blogs; and public statements. The literature research was key in establishing a selection criterion for experts in the study, to guide us in the design of the interview topic guide, to feed our study while we were conducting fieldwork, and to triangulate information gathered in the interviews.

We conducted in-depth interviews between January and March 2021 to understand the ideas held by experts regarding COVAX and access to COVID-19 vaccines at the global and national levels. We used a purposeful selection strategy for sampling: participants were selected deliberately in order to provide us with relevant information that we could not have accessed through direct observation nor by other methodological choices [10].

Table 1 shows relevant features of our panel of twelve interviewees who hold senior roles in their institutions and who either have expertise in global access to COVID-19 vaccines or are privileged observers in the process of vaccine procurement in specific countries. Our study participants are experts directly involved in the COVAX scheme, as well as experts from academia; the World Health Organization; and NGOs specialised in access to medicines and intellectual property rights. Additionally, we interviewed public officials from health ministries and other relevant public divisions to obtain COVAX-related information from high-income countries (Norway and Switzerland), and middle-income countries (Argentina and Kazakhstan) [11]. We intentionally did not include experts from low-income and lower-middle-income countries. As these are “funded” countries, they are eligible to participate in the COVAX scheme supported by the COVAX Advance Market Commitment [12], and thus don’t require any incentives other than receiving free vaccines. It is unlikely that they will sign APAs to buy vaccines, however, they may access vaccines through Gavi.

**TABLE 1:** Interviewees’ expertise, institution, role, and region. Own creation, Switzerland, 2021.

Interviewee	Expertise	Institution	Role	Region where expert is based
Expert 1	Country level	Academia/Institute of Public Health	Researcher/Policy Officer	Europe
Expert 2	Country level	Institute of Public Health	Policy Officer	Latin America
Expert 3	Country level	International Health Organization	Policy Officer	Asia
Expert 4	Country level	Ministry of Health	Politician	Latin America
Expert 5	Country level	Ministry of Health	Policy Officer	Europe
Expert 6	Global level	Academia/International Health Organization	Researcher/Consultant	Europe
Expert 7	Global level	International Health Foundation	Chief Executive Officer	Europe
Expert 8	Global level	International Health NGO	Manager	Europe
Expert 9	Global level	International Health Organization	Public Health Expert	Asia
Expert 10	Global level	International Health Organization	Epidemiologist	Latin America
Expert 11	Global level	International Health Think Tank	Executive director	Europe
Expert 12	Global level	International Political Organization	Policy Officer	Europe

The topic interview guide complies with the standards for expert interviews [13]. Interviews were guided by the following topics: the current design and relevance of COVAX; its incentives, functioning and implementation; vaccine development and production; and the challenges to equitable access to COVID-19 vaccines. Interviews were adapted according to the stakeholders; i.e., when interviewing COVAX experts, there was greater emphasis on the scheme itself, and when interviewing country officials, special attention was paid to country perspectives, incentives and challenges. However, the topics we discussed with all interviewees were the same. We built a data analysis matrix [10] based on the interview transcripts and conducted a thematic analysis.

## Results

In this section we present the findings from expert interviews regarding the relevance of COVAX, the varied incentives to join the scheme, and implementation issues in the early phase of COVAX.

### Relevance of COVAX

One primary finding is that experts who sustain different positions regarding equitable access to COVID-19 vaccines spoke in high terms of the COVAX scheme. Some of the benefits associated with COVAX, as identified by interviewees, include; having access to the greatest variety of vaccines available, fair prices, the fact that COVAX enables cost-sharing, and a better access to vaccines for low- and middle-income countries. Experts also emphasised that the scheme has particularly helped create more transparency regarding the safety and effectiveness of vaccines by establishing a pre-qualification mechanism. COVAX is furthermore perceived by some experts as a praiseworthy undertaking, as the best cooperative scheme that has been achieved in history of global vaccine distribution, and as a sustainable mechanism in the actual circumstances of a complex and imperfect world. In the view of interviewees, COVAX is taking place in a world dominated by market laws and opacity about decisions, characterised by unequal bargaining situations of different countries, geopolitical conflicts, fake news and a general lack of willingness to cooperate at the global level.

### Incentives to Join the COVAX Scheme

#### Access to Vaccines

Experts from governments of consulted upper-middle-income countries are primarily interested in the vaccines that COVAX distributes and in getting associated technical support, which is not the case regarding high-income countries. One relevant finding is that according to some of the experts, the main designed incentive to join COVAX—the access to safe and effective vaccines—is secondary for some high-income countries. In contrast, public health experts consulted in upper-middle-income countries indicated that getting access to vaccines for up to 20% of the population is the major incentive for joining COVAX. The scheme would allow countries to access a broad portfolio of vaccines, and avoid co-dependencies from bilateral manufacturers. For instance, Argentinian experts claimed that Argentina would be given the opportunity to access vaccines other than Sputnik V, in a context in which other bilateral negotiations had not been successful (e.g., with Pfizer). Experts in Kazakhstan (the only country in our case study that is not part of COVAX), similarly expressed that if they joined the scheme, they would not depend exclusively on the delivery of Sputnik V. At the time of the interviews, there was no evidence yet of a phase III trial to support Sputnik V, and the vaccine had not yet been approved by major international regulatory agencies. Later this year, evidence showed that Sputnik V was a safe and effective vaccine [14].

#### Foreign Health Policy

A major incentive for some high-income countries to join COVAX is to consolidate their foreign health policy. However, this finding is limited to expert opinions from two countries; Norway and Switzerland. According to these experts, the development and consolidation of Swiss foreign health policy and health diplomacy are very important because the country hosts several international health organizations. The interviewee based in Norway pointed out similar considerations, that Norway is interested in maintaining its image as a pioneering country in the global health movement and as one of the important funding partners of Gavi. It remains to be investigated whether other high-income countries participating in COVAX follow similar incentives.

#### Biosecurity

Experts from upper-middle and high-income countries further emphasise biosecurity as a strong reason for promoting COVAX. Some of them emphasise that a major concern is indeed that the well-being of rich countries depends on the well-being of the global poor. According to these interviewees, the COVAX scheme has a collective global interest to stop virus circulation and mutation genesis.

### Implementation Issues in the Early Phase of COVAX

#### Funding

Experts that hold different views regarding who should get priority access to COVID-19 vaccines, stress that it is unclear whether the current funding of COVAX is sufficient to achieve the goal of delivering two billion vaccines in 2021. Moreover, several experts mentioned a high degree of uncertainty and lack of transparency, that would be required to determine whether funding is sufficient. One expert pointed out that initial payments for APAs were done as anticipated, but given the uncertainty of supply shortage, the approval of vaccine candidates in the portfolio, and production volatility, it is hard to assess whether funding is enough. One expert that could report on direct insights of the COVAX facility, stated that funding is a major problem and that high-income countries are financing the scheme to soothe their conscience, but the money is not enough to make it a sustainable enterprise. Relatedly, some experts also argued that the funding provided could have been used more effectively. For instance, instead of investing in the development of vaccines (e.g., AstraZeneca), COVAX could have secured more APAs with other manufacturers, given that vaccine development was greatly incentivised because of high global demand.

#### Vaccine Production, Technology Transfer and Intellectual Property

Experts consulted believe that a better technology transfer is needed to scale up vaccine production. However, experts hold heterogenous opinions regarding compulsory licensing (CL) and waivers of intellectual property rights (IP) as legal tools to enable technology transfer. COVAX has promised to scale up vaccine manufacturing, but some experts complain that little has happened so far. They perceive that not enough vaccines are being produced, and this is one of the most pressing concerns in the implementation of COVAX. In many countries, there is a know-how to manufacture vaccines and some experts agree that these capacities need to be used more efficiently (e.g., by supporting technology transfer between innovators and production sites in more regions).

Experts hold different views on whether compulsory licensing (CL) or a waiver of intellectual property (IP) rights would help accelerate technology transfer and scale up production. CL is perceived by some experts as a tool that can indeed help facilitate the production of vaccines. Others think that it is useless in view of the goal to increase manufacturing capacities. A third group perceives CL as counterproductive, stating that it might destroy trust and incentives given to the pharmaceutical companies in the long run. Some experts claimed that a waiver of IP rights and CL is obsolete, and that the debate about CL and a waiver of IP rights is of ideological nature. These experts also believe that it would be useful to discuss how pharmaceutical innovators and manufacturers could cooperate under license agreements, as shown by the successful partnership between the Serum Institute in India and AstraZeneca, who currently produce more than 70 million doses per month [15].

#### Obstacles to Multilateralism

Some experts agree that multilateralism is essential to the success of COVAX, although they disagree on the degree of realisation of multilateralism. Some experts state that a truly multilateral approach to global health and vaccine distribution is missing. Based on the theoretical assumption that global vaccine supply resembles a zero-sum-game, some experts say that an international agreement is necessary to enforce caps on bilateral deals, which would prevent competition between COVAX and rich countries for access to vaccines. A zero-sum game represents a situation in which one participant’s gain or loss of utility is balanced out by the loss or gain of utility from other participants. If the total gains of the participants are added up and the total losses are subtracted, they will sum to zero. However, other experts suggest that bilateral deals and major pre-purchasing commitments have considerably accelerated vaccine research and development. They claim that low- and middle-income countries have hugely benefited from this, and that COVAX would have never managed to achieve this on its own.

#### Distribution Principle and Proportional Access

Some experts hold the view that proportional distribution is a good first step in an emergency situation, but alternative principles and allocation schemes should be further discussed during the implementation of COVAX. Interviewees perceived the proportional distribution principle of 20% to be a good factor in convincing a variety of countries to join COVAX, including some self-financing countries. However, some experts suggest that the proportionality principle should be reassessed because a proportional distribution scheme ignores needs-based considerations. For example, to achieve efficient implementation of resources in terms of health impact, evaluations would be necessary to determine which countries are most harmed by the virus. In practice, some experts agree that wealthier countries that have signed bilateral agreements and/or advanced their vaccination campaigns at a fast pace, will most likely forgo their share of COVAX vaccines.

#### Vaccine Portfolio

Some experts suggest that COVAX would do better when expanding its vaccine portfolio and including more promising vaccine candidates that are still in the pipeline. These experts also call for improved transparency of candidates that are already included in the portfolio and also those which are soon to be integrated. This idea was not shared by one of the experts, who posed that the current vaccine portfolio constitutes an excellent representation of successful vaccine candidates.

## Discussion

### Relevance of COVAX

COVAX is a middle-ground strategy for cooperation, where countries can still buy vaccines outside the scheme, and where there are no international agreements on caps regarding bilateral deals. Interestingly, while the interviewed experts hold different views on vaccine allocation, they agreed on the importance of joining a global cooperation mechanism like COVAX. This is relevant for demonstrating solidarity and consolidating COVAX’s attempts to reconcile the interests of those who believe that a person’s nationality should not affect their access to life-saving interventions, such as the COVID-19 vaccine, and the interests of those who defend to prioritise the vaccination of fellow citizens.

In the broader context of vaccine allocation in global emergencies, COVAX demonstrates an improved global response when compared to the H1N1 influenza virus in 2009. There were approximately 300,000 deaths from the H1N1 outbreak, even though a vaccine had been developed within 7 months from the beginning of the pandemic. At that time, 90% of the total vaccine production was made accessible to ten countries that could afford them. After an intervention by the WHO, 10% of the vaccine doses were then made accessible to other countries [7].

### Incentives to Join COVAX

Our findings show that incentives revealed by experts based in upper-middle income countries are congruent with the incentives foreseen by COVAX. These experts pointed that major incentives are to gain access to 1) a well-developed vaccine portfolio, 2) an insurance mechanism; and 3) the spillovers that are created in the course of global efforts to produce vaccines (e.g., know-how of failed vaccines that can be further used for the development of an alternative vaccine candidate). This is further supported by the demand of countries to join COVAX through commitment agreements and confirmations of intent to participate [12].

Contrary to the assumption that high-income countries would be mainly interested in COVAX for its design, empirical findings show that at least for some high-income countries, the main incentive is to position themselves as relevant international actors in global health. From a conceptual point of view, this is particularly interesting as high-income countries might have an incentive to donate vaccine doses directly to low- or middle-income countries to gain geopolitical influence (without necessarily joining COVAX). Furthermore, both the design of COVAX and the experts’ views coincide on biosecurity—understood as stopping virus circulation—as an incentive to join the scheme.

High- and middle-income countries have additional economic incentives to join COVAX. A study by the ICC Research Foundation estimates losses of USD 9.2 trillion globally—of which USD 4.5 trillion would be losses from wealthy countries—if we fail to ensure lower income economies’ access to COVID-19 vaccines [16]. Additionally, accessing vaccines through COVAX might prevent middle-income countries from paying for overpriced vaccines. For instance, South Africa secured one million doses of the AstraZeneca vaccine at a price of USD 5.25 per dose at the end of January, while European Union countries paid USD 2.16 for the same, a difference of more than half the price. South Africa was unable to secure a better pricing agreement even though the country hosted a clinical trial of the AstraZeneca vaccine, which violates the principles of post-trial access and fair benefit sharing with communities [17].

### Implementation Issues

Even if experts agree on the importance of multilateralism in the success of COVAX, there is no agreement on its implementation, in particular regarding bilateral deals and pre-purchasing commitments. Currently, COVAX is underfunded and bilateral deals are proliferating [18]. In the early implementation phase of COVAX, countries seemed to be trapped in a zero-sum game regarding the global vaccine supply. The theoretical analysis in which bilateral deals could be globally beneficial due to an expansion of the development and production of vaccines is currently not applicable. This analysis worked well during the earlier stages of vaccine development, however, bottlenecks are appearing in vaccine manufacture, technology transfer and intellectual property.

COVAX’s proportional distribution principle of vaccines, in which participating countries get vaccines for 20% of their population, is considered by many experts as a good first step in the pandemic. However, from a conceptual perspective, the end-state of vaccine distribution is not the only matter of relevance, but also the allocation procedure itself. Some problems have already emerged. For instance, in February 2021, COVAX allocated its first shipment of vaccines to Ghana, a country with 30 million inhabitants, 86,000 total cases of COVID-19, and 700 deaths caused by the virus. Comparatively, Peru has a similar population of 32 million inhabitants, but had 1.4 million cases of COVID-19 and 48,000 associated deaths at the same time [19]. The fair priority model advocates a needs-based distribution instead of a proportional equity criterion [20]. Under a model like this, Peru would have been given priority over other countries whose situation was less desperate at the time.

### Limitations

We would like to note some limitations of this study. One selection problem that we face is the key informant bias [10]. Our study relies on a small number of participants and even if they were purposefully selected and provided rich data, there is no guarantee that our participants’ views are typical, and we cannot assume that their discourses incorporate enough diversity and homogeneity [10]. The findings of the interviews are limited to a cohort of twelve experts and may not fully represent the wider expert community. Nonetheless, since we gathered experts who hold the widest possible range of views on COVAX and equitable access to vaccines, we had access to the entire spectrum. We reached theme saturation for all topics covered in our interview guide. Additional data provided after our ninth interview did not lead to emergent themes, and we conducted three more interviews to ensure that the new data was repeating what had been expressed by other interviewees. Another issue is that the sources that we cite in this report, as well as the empirical findings from the interviews, need to be contextualised within the specific period of data collection. We conducted the interviews between late January and March 2021, at a time when COVAX started allocating vaccines. It is likely that the experts’ views may change due to the quick development of the pandemic, the ongoing implementation of COVAX and vaccine delivery, together with technical advancements and unforeseen novelties.

### Conclusion

This study contributes to the debate on equitable access to COVID-19 vaccines by providing empirical findings that enrich ethical discussions, and highlighting policy challenges regarding the relevance and design of COVAX; the heterogenous incentives to join the scheme; and the implementation issues that have emerged in COVAX’s early phases.

Now that more than 6 months have passed since we conducted the interviews for this study, COVAX has shipped around 330 million vaccines to 144 countries, but the two billion dose target for 2021 will be missed and 98% of people in low-income countries remain unvaccinated [21]. The findings and discussions we present here may serve as inputs for later stages of COVAX and for the design and development of future global cooperation mechanisms for public health emergencies.

We offer novel insights on three ethical discussions that provide a common ground for equitable access to COVID-19 vaccines. First, the design of COVAX offers a concrete mechanism to advance a fairer global vaccine allocation by reconciling the interests of moderate vaccine nationalists and cosmopolitans. Second, the incentives to join COVAX may respond to self-interest (e.g., access a pool of safe and effective vaccines, strengthen countries’ roles as prominent global health actors), to solidarity (e.g., decrease suffering and death globally), or both (e.g., biosecurity). Third, although COVAX’s proportional distribution principle promotes equitable access as a final goal, it remains to be discussed how we can ensure that the process of global vaccine allocation is also fair.

We also present and discuss some key policy challenges that emerged in the early implementation phase of COVAX. Additional principles to further advance equitable access might be implemented to ensure that countries who face a more urgent situation regarding the pandemic are prioritised in the allocation of vaccines by COVAX. We also highlight that some countries joined the scheme pursuing interests that were different from the ones foreseen by COVAX and could, therefore, be emphasised in later stages. Lastly, some key implementation challenges in the early phase of COVAX are: the securing of funding; the improvement of vaccine production and technology transfer; the establishment of further discussions on intellectual property; countries finding themselves in a zero-sum game that hinders global vaccine supply; and the potential expansion of the vaccine portfolio.

### Remarks on Interviewee Participation and Literature Research

All experts participated in the study voluntarily and pro bono. We conducted the interviews online in Spanish, English, or German. Interviews lasted between 35 and 100 min, and most of them were audio-recorded (the interviewer took notes when participants did not want to be recorded). The transcriptions of audio recorded interviews were done by the authors of this paper in the same language in which they were conducted. We conducted the thematic analysis and the data analysis matrix model in English. The names and positions of the interviewees are not revealed and data was handled and stored in accordance with data protection rules of the University of Zurich.

The literature research was mainly carried out in January 2021, although we monthly sought for new relevant texts until May 2021. We searched for academic articles published in English in PubMed, Google Scholar and specialised journals using the keywords “ethics,” “COVAX,” “Covid-19 vaccines,” “fair distribution,” “equitable distribution,” “global access,” “nationalism,” and ‘cosmopolitanism.” We sought media reports in newspapers and blogs, and for public statements in English, German and Spanish. Through the literature research we mapped key institutions and roles in global access to COVID-19 vaccines and vaccine procurement at the national level, we gained detailed information about the incentives, functioning and implementation of COVAX, as well as common challenges to equitable access to health goods in emergency settings, and we were able to give better context and examples to our interviewees.
